# Supplementary material for: Identification of photoperiod-induced specific miRNAs in the adrenal glands of Sunite sheep (Ovis aries)
Source: Front Vet Sci. 2022 Jul 22;9:888207. doi: 10.3389/fvets.2022.888207 (PMC9354845; doi:10.3389/fvets.2022.888207)
Supplement: Supplementary file 1 [file Data_Sheet_1.ZIP › Supplementary file/miRDeep2 file.zip!/miRDeep2 file/Novel_Structure/LP42A1/Novel_498.pdf]

Provisional ID	:	NC_019463.2_13900
Score total	:	1.6
Score for star read(s)	:	-1.3
Score for read counts	:	-0.8
Score for mfe	:	2.2
Score for randfold	:	1.6
Score for cons. seed	:	
Total read count	:	10
Mature read count	:	10
Loop read count	:	0
Star read count	:	0

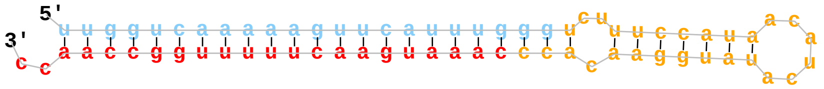

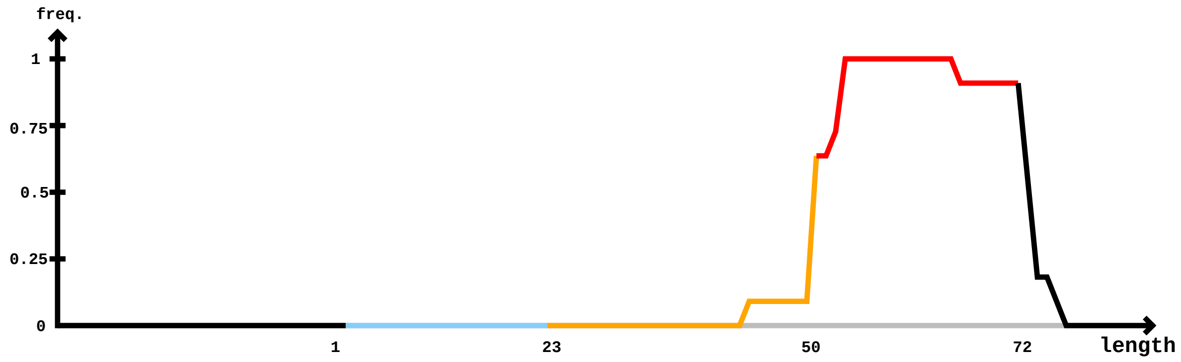


```
                         Star                                  Mature
5'- aaagcauucuuuuauguccacuaauagguuggucaaaaaguucauuugggucuuuccauaacaucauauggaacacccaaaugaacuuuuuggccaacccauacaucauaca   -3'    exp
    ...((((......))))........(((((((((((((((((((((((((..(((((((......))))))).)))))))))))))))))))))))))).............     reads      mm         sample
    ......................................................................gaacacccaaaugaacuuuuug....................     1          0          seq
    .............................................................................caaaugaacuuuCuggccaacc.............     5          1          seq
    .............................................................................Uaaaugaacuuuuuggccaacc.............     1          1          seq
    ...............................................................................aaugaacuuuuuggccaacc.............     1          0          seq
    ................................................................................augaacuuuuuggccaaccc............     1          0          seq
    ................................................................................augaacuuuuuggccaacGcau..........     1          1          seq
    ................................................................................augaacuuuuuggccaacccaGa.........     1          1          seq
```
